# Cell proliferation by silk gut incorporating FGF-2 protein microcrystals

**DOI:** 10.1038/srep11051

**Published:** 2015-06-08

**Authors:** Eiji Kotani, Naoto Yamamoto, Isao Kobayashi, Keiro Uchino, Sayaka Muto, Hiroshi Ijiri, Junji Shimabukuro, Toshiki Tamura, Hideki Sezutsu, Hajime Mori

**Affiliations:** 1 Department of Applied Biology, Kyoto Institute of Technology, Sakyo-ku, Kyoto 606-8585, Japan; 2 Insect Biomedical Centre, Kyoto Institute of Technology, Sakyo-ku, Kyoto 606-8585, Japan; 3 Transgenic Silkworm Research Unit, National Institute of Agrobiological Sciences, Tsukuba, Ibaraki 305-8634, Japan

## Abstract

Silk gut processed from the silk glands of the silkworm could be an ideal biodegradable carrier for cell growth factors. We previously demonstrated that polyhedra, microcrystals of Cypovirus 1 polyhedrin, can serve as versatile carrier proteins. Here, we report the generation of a transgenic silkworm that expresses polyhedrin together with human basic fibroblast growth factor (FGF-2) in its posterior silk glands to utilize silk gut as a proteinaceous carrier to protect and slowly release active cell growth factors. In the posterior silk glands, polyhedrin formed polyhedral microcrystals, and FGF-2 became encapsulated within the polyhedra due to a polyhedron-immobilization signal. Silk gut powder prepared from posterior silk glands containing polyhedron-encapsulated FGF-2 stimulated the phosphorylation of p44/p42 MAP kinase and induced the proliferation of serum-starved NIH3T3 cells by releasing bioactive FGF-2. Even after a one-week incubation at 25 °C, significantly higher biological activity of FGF-2 was observed for silk gut powder incorporating polyhedron-encapsulated FGF-2 relative to silk gut powder with non-encapsulated FGF-2. Our results demonstrate that posterior silk glands incorporating polyhedron-encapsulated FGF-2 are applicable to the preparation of biodegradable silk gut, which can protect and release FGF-2 that is produced in a virus- and serum-free expression system with significant application potential.

Cypoviruses belong to a genus within the *Reoviridae* family, and their infection of insect larvae is characterized by the production of massive numbers of virus-encoded protein polyhedra^1^. The polyhedrin protein molecules crystallize in the cell cytoplasm to form cubic inclusion body polyhedra that encapsulate numerous viral particles. Polyhedra function to facilitate infection from one insect host to another by allowing long-term viability of the stabilized virions in hostile environments^2^.

We have developed targeting strategies using polyhedron-immobilization signal sequences to encapsulate foreign proteins within Cypovirus 1 polyhedra^3^,^4^. Previous studies have demonstrated that polyhedra can artificially regulate cell proliferation as versatile protein microcrystal carriers of cell growth factors. Polyhedra enable this regulation both by protecting proteins against desiccation for long periods and by slowly releasing proteins from the protective envelope^3^,^4^,^5^,^6^. Previous examples have included polyhedra encapsulating human basic fibroblast growth factor (FGF-2), which showed mitogenic activity by stimulating the proliferation of mesenchymal cells including NIH3T3 fibroblasts and ATDC5 cells^3^,^4^. Clinically, similar approaches have accelerated scald and wound healing^7^.

We have established a system to create cell growth factor-encapsulating polyhedra by using insect cell culture accompanied by mammalian foetal serum and baculovirus vector systems. For cell growth factor-encapsulating polyhedra to be clinically applicable, polyhedra should be produced in a virus-free and serum-free system. However, no studies have investigated whether cell growth factor-encapsulating polyhedra can be produced in *in vivo* eukaryotic cell systems without utilizing a viral vector. Methods for generating transgenic silkworms have been established: one uses a *piggyBac* transposon, and another uses a baculovirus-based vector with a targeting sequence for the silkworm genome^8^,^9^,^10^,^11^,^12^. Accordingly, transgenic silkworms have been studied as tools for the massive production of foreign recombinant proteins because silkworm silk glands have high protein biosynthesis capacity. Moreover, the *GAL4/UAS* system has been adapted for transgenic silkworms, thereby allowing the generation of transgenic lines expressing a foreign protein specifically in a certain tissue, for example in the posterior silk gland^13^,^14^,^15^.

A wide range of organic and inorganic materials have been tested as cell culture matrices together with cell growth factors to stimulate cell proliferation both *in vitro* and *in vivo*, among them silk fibroin^16^,^17^,^18^ produced in the *Bombyx mori* silk gland. Physical and chemical manipulation of *B. mori* silk fibroin has resulted in silk matrices suitable for cell proliferation that are both biocompatible and predictably degradable over the long term^19^,^20^. The *Bombyx* silk gland is physically processed into silk guts for use as fishing line, and it is also suitable for efficient expression of foreign proteins that should be produced in a virus-free and serum-free environment. In addition, the *Bombyx* silk gland is a versatile matrix for artificial control of cell proliferation. In the present study, we generated a transgenic silkworm line with posterior silk glands that express polyhedron-immobilization signal-conjugated FGF-2 and polyhedrin which mainly crystallizes at the spinning stage of the 5th instar. The posterior silk glands were physically processed into silk guts and further crushed into fine silk gut powder. Furthermore, we showed that silk gut powder including polyhedron-encapsulated FGF-2 demonstrated FGF-2 release into a culture of serum-starved NIH3T3 cells leading to cellular MAP kinase activation and cell proliferation. Moreover, polyhedra in silk gut powder were able to stabilize encapsulated-FGF-2, even after incubation for one week at 25 °C. These experiments demonstrate how silk guts can be used to provide bioactive and stable FGF-2 that can control cells long-term both *in vivo* and *in vitro*.

## Results

**Figure 1:**
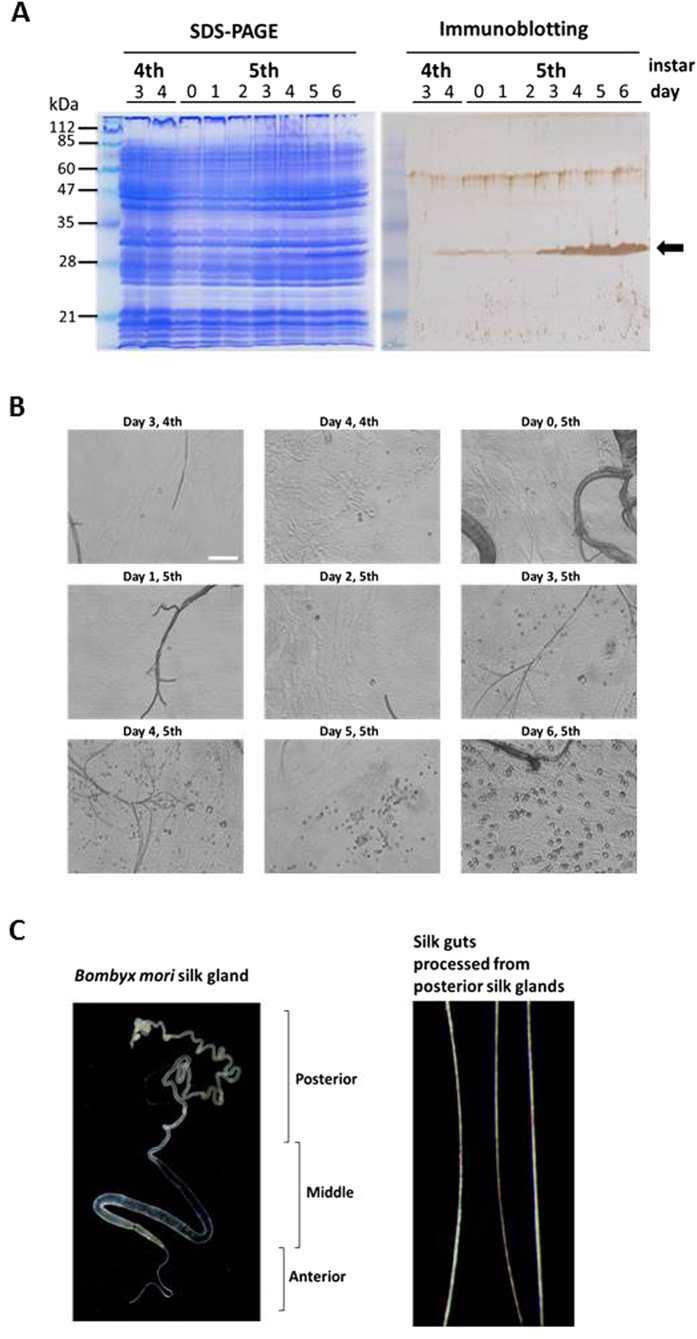
Polyhedrin expression and crystallization in posterior silk glands. (**A**) Detection of polyhedrin in posterior silk glands. Protein samples (20 μl) of posterior silk glands from the BmFibH-polyhedrin line on the indicated days of the 4th and 5th instars were electrophoresed in a 12.5% SDS-PAGE gel with a molecular marker (left panel) and transferred onto nitrocellulose membrane (right panel). Polyhedrin (arrow) was detected by immunoblotting as described in the Methods section. (**B**) Polyhedra formed in the posterior silk glands of the BmFibH-polyhedrin line were observed under a light microscope with the objective lens (20x). Bar, 25 μm. (**C**) A silk gland dissected from *w1-pnd* strain (left panel) and silk gut physically processed from the posterior silk glands after air drying (right panel).

We firstly asked whether Cypovirus 1 polyhedrin could be practically expressed and crystallized to form polyhedra in the posterior silk gland. A transgenic line, BmFibH-polyhedrin, was generated using strategies described in the Supplementary Methods (Supplementary Information). We established an intermediate UAS-polyhedrin line that carries the UAS-polyhedrin gene, a transgene from the vector pBacMCS[A3KMO,UAS-polyhedrin-SV40] vector (Supplementary Fig. S1A). The inverse-PCR method determined that the transgene locus of the UAS-polyhedrin line is in chromosome 6 (Supplementary Table S1). Next, a BmFibH-polyhedrin line was generated by mating the UAS-polyhedrin line with a BmFibH-GAL4 line carrying the GAL4 gene controlled under the fibroin-heavy chain (fibroin H) promoter, as described previously^15^. Thus, BmFibH-polyhedrin is designed as a line expressing polyhedrin protein exclusively in the larval posterior silk gland concomitantly with fibroin H expression. Posterior silk gland proteins were analysed by immunoblotting during the 4th and 5th instar developmental stages (Fig. 1A). In the present study, larvae of the BmFibH-polyhedrin line initiated spinning on the 5th day in the 5th instar, and on the 6th day, the larvae continued spinning. Immunoblotting with anti-polyhedrin antibody revealed polyhedrin protein at every stage (Fig. 1A, right panel); polyhedrin was strongly expressed, especially from the 3rd to 6th day in the 5th instar, and from the 4th to 6th day polyhedrin production appeared to reach its maximum. It is known that GAL4 protein expressed under control of the fibroin H promoter is initially detected especially in the posterior silk glands on the 3rd day of 5th instar larvae, and strong expression of GAL4 has been observed only after a later stage of the 5th instar^15^. Taken together, our initial results indicated that polyhedrin was expressed in the silkworm’s posterior silk glands, and the expression pattern correlated well with known pattern of expression of the fibroin H gene promoter.

To investigate the formation of polyhedron crystals in the BmFibH-polyhedrin line, posterior silk glands were excised from the larvae during the 4th and 5th instar developmental stages, and then the crushed tissue was observed on glass slides under a light microscope (Fig. 1B). Typical polyhedral crystal structures were clearly observed in the tissue. The size and number of the crystals rose drastically on the 6th day of the 5th instar (Fig. 1B). These observations clearly demonstrated that polyhedrin forms crystal structures in eukaryotic tissue, the posterior silk gland, as it does in insect culture cells.

Silk guts (Fig. 1C, right panel) processed from posterior silk glands of the silkworm (Fig. 1C, left panel) could be biodegradable and serve as ideal carriers for cell growth factors that control cellular proliferation. However, no studies have investigated whether silk guts incorporating polyhedra, versatile protein microcrystal carriers of cell growth factors, enable the regulation of cell proliferation both by protecting functional proteins against hostile environments and by slowly releasing proteins^3^,^4^,^5^,^6^. The present study aimed to generate a transgenic silkworm with functional silk glands that produce and incorporate active FGF-2 encapsulated within polyhedra in a serum- and virus-free system.

Our previous studies determined that the H1-helix portion of polyhedrin functions as a polyhedron-immobilization signal and is used to target foreign proteins into polyhedra in the insect cell culture system^3^. To elucidate whether the H1 signal functions to encapsulate proteins into polyhedra in this system, we established the transgenic line BmFibH-polyhedrin/H1/FGF-2 (Supplementary Fig. S1B). An intermediate line, UAS-H1/FGF-2, was established using the vector pBacMCS[UAS-H1/FGF-2-SV40,3xP3-GFP] (Supplementary Fig. S1A) carrying human FGF-2 with the polyhedrin H1 sequence fused to its N-terminus (H1/FGF-2)^3^. The inverse-PCR method confirmed that the transgene sequence from the vector was inserted into silkworm chromosome 11 (Supplementary Table S1). Then, the UAS-H1/FGF-2 line was mated with the BmFibH-polyhedrin line to generate the BmFibH-polyhedrin/H1/FGF-2 line designed to express both polyhedrin and H1/FGF-2 under control of the fibroin H promoter, as represented in Supplementary Figure S1B. A BmFibH-H1/FGF-2 line, designed to express only H1/FGF-2 under control of the fibroin H promoter exclusively in posterior silk glands, was generated by mating the UAS-H1/FGF-2 line with the BmFibH-GAL4 line (Supplementary Fig. S1C).

**Figure 2:**
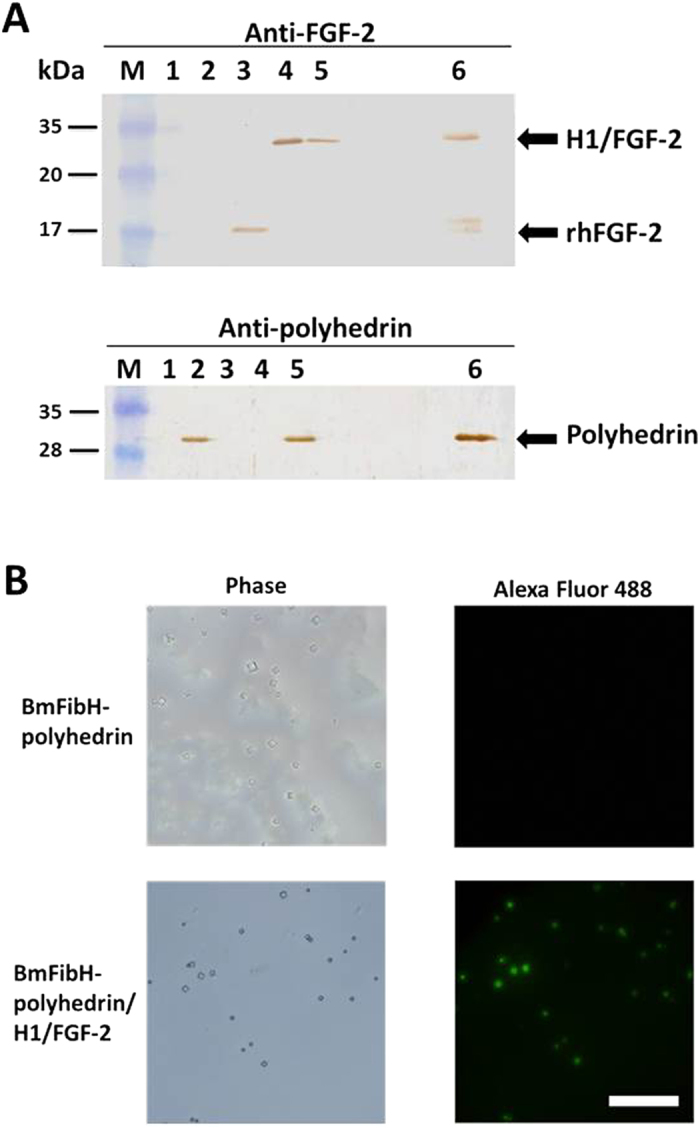
Encapsulation of FGF-2 within polyhedra in the posterior silk glands of transgenic silkworms. (**A**) Detection of the recombinant protein in posterior silk glands. Protein samples (7 μl) from *w1-pnd* strain (lane 1), BmFibH-polyhedrin line (lane 2), BmFibH-H1/FGF-2 line (lane 4), and BmFibH-polyhedrin/H1/FGF-2 line (lane 5) posterior silk glands on the 6th day of the 5th instar were electrophoresed in a 12.5% gel with a molecular marker (lane M), rhFGF-2 (lane 3), and polyhedron-encapsulated H1/FGF-2 prepared from cultured Sf21 cells (lane 6); FGF-2 (upper panel) and polyhedrin (lower panel) were detected by immunoblotting using anti-polyhedrin and anti-FGF-2 antibodies as described in the Methods section. Degraded H1/FGF-2 bands (approximately 17 kDa) were observed in lane 6 (upper panel). Arrows indicate polyhedrin, H1/FGF-2, and rhFGF-2. Original gel images of the data are presented in Supplementary Figure S2. (**B**) Polyhedra formed in the posterior silk glands of BmFibH-polyhedrin and BmFibH-polyhedrin/H1/FGF-2 lines were fixed on a glass-based dish and the H1/FGF-2 encapsulated within the polyhedra was detected by immunofluorescence as described in the Methods section. Bar, 50 μm.

Posterior silk glands were excised from the transgenic lines on the 6th day of the 5th instar, and their proteins were analysed by immunoblotting with anti-polyhedrin or anti-FGF-2 antibody (Fig. 2A). Commercial human recombinant FGF-2 (rhFGF-2) and polyhedron-encapsulated H1/FGF-2 prepared from the Sf21 cell culture system^3^ were also applied to the immunoblots as positive controls. Posterior silk glands from non-transgenic *w1-pnd* were analysed as a negative control. H1/FGF-2 was detected in the posterior silk glands from the BmFibH-polyhedrin/H1/FGF-2 and BmFibH-H1/FGF-2 lines, as were the positive control proteins rhFGF-2 and polyhedron-encapsulated H1/FGF-2 from Sf21 cells (Fig. 2A, upper panel). The immunoblotting also revealed that the posterior silk glands of the BmFibH-polyhedrin/H1/FGF-2 line efficiently express polyhedrin protein (Fig. 2A, lower panel). To determine whether the H1/FGF-2 was encapsulated within crystallized polyhedra in the posterior silk glands of the BmFibH-polyhedrin/H1/FGF-2 line, polyhedra from the posterior silk glands suspended in PBS buffer were fixed on a glass-based dish, and immunofluorescence was analysed using anti-FGF-2 (Fig. 2B). Fluorescent anti-FGF-2 signals were detected on polyhedra from posterior silk glands of the BmFibH-polyhedrin/H1/FGF-2 line, but they were not detected from posterior silk glands of the BmFibH-polyhedrin line used as a control, although they were similarly treated with the antibody (Fig. 2B). These results indicated that H1/FGF-2 is encapsulated within polyhedra formed in the posterior silk glands.

**Figure 3:**
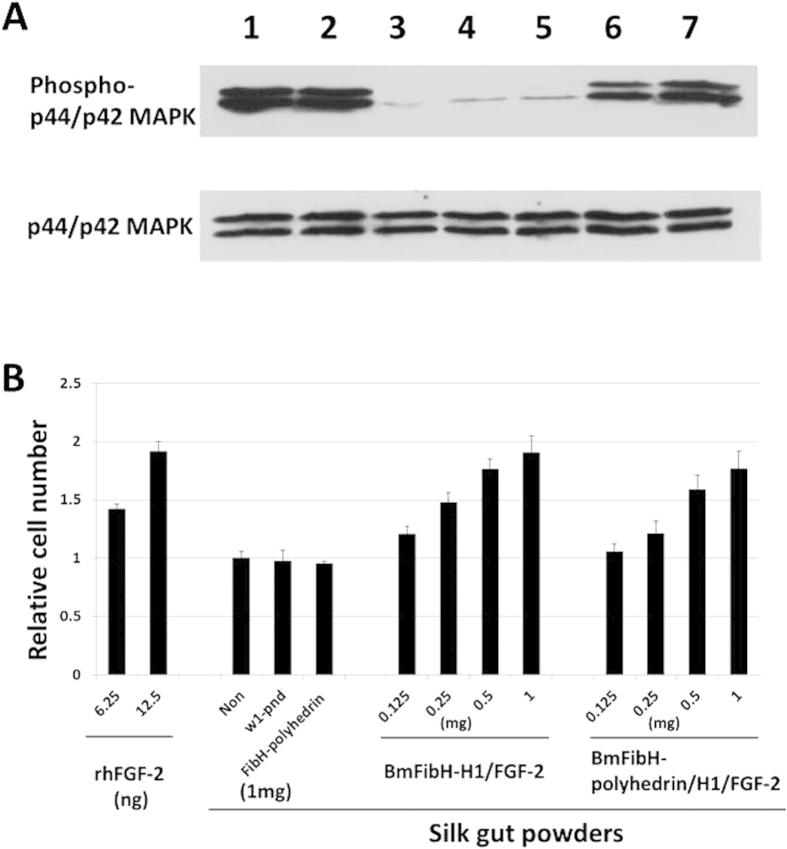
Proliferation of NIH3T3 cells induced by silk gut powder from transgenic silkworms. (**A**) Detection of phosphorylated p44/p42 mitogen-activated protein kinase (MAPK) in NIH3T3 cells. Silk gut powders (1 mg/well) from the *w1-pnd* strain as a non-transgenic negative control (lane 4), from the BmFibH-polyhedrin line as a transgenic negative control (lane 5), and from lines BmFibH-H1/FGF-2 (lane 6) and BmFibH-polyhedrin/H1/FGF-2 (lane 7) were placed on the inside filters of cell culture inserts, which were then introduced into the medium of serum-starved NIH3T3 cells. Additionally, cells were not treated (lane 3; negative control) or similarly treated with 10 ng/well of rhFGF-2 (positive control; lane 1) or 1 × 10^5^ cube/well of H1/FGF-2-encapsulating polyhedra from Sf21 (positive control; lane 2). After 4 h of cultivation, cell lysate samples (5 μg proteins) were analysed by immunoblotting using either p44/p42 MAPK antibody or phosphorylated p44/p42 MAPK antibody. Original gel images of the data are presented in Supplementary Figure S2. (**B**) Relative cell numbers were measured by WST-8 assay. After starvation, cells were cultured in assay medium containing either rhFGF-2 or cell culture inserts containing the indicated amounts of silk gut powders from *w1-pnd* strain, BmFibH-polyhedrin line, BmFibH-polyhedrin/H1/FGF-2 line or BmFibH-H1/FGF-2 line. After incubation for 48 h at 37 °C under 5% CO_2_, cell numbers were measured by WST-8 assay. Absorbance at 450 nm was determined by using a micro-plate reader and mean values of triplicate experiments are presented with standard deviations.

To assess the biological function of H1/FGF-2 produced in the posterior silk glands, silk guts were processed from posterior silk glands as depicted in Fig. 1C. The silk guts were further lyophilized and crushed into fine powdery material (silk gut powder) and stored at −80 °C. It is well known that intracellular signalling events such as receptor-mediated phosphorylation of p44/p42 mitogen-activated protein kinase (MAPK) are observed after FGF-2 stimulation^21^,^22^,^23^. We previously reported that the introduction of polyhedron-encapsulated FGF-2 prepared from the Sf21 cell culture induced phosphorylation of p44/p42 MAPK^3^,^4^. Silk gut powder was placed on the inside filter of a cell culture insert (see the Methods section) and then introduced into the medium of serum-starved NIH3T3 cells. The cells were incubated with the silk gut powder for 4 h, and the phosphorylation of p44/p42 MAPK was observed by using a phospho-p44/p42 MAPK specific antibody (Fig. 3A). Sufficient phosphorylated p44/p42 MAPK could be detected only when active FGF-2 was released from the silk gut powder, diffused into the medium, passed through the inside filter of the cell culture insert and stimulated the cell proliferation because the cells did not directly contact the silk gut powder but were approximately 2 mm distant from it. We found that H1/FGF-2 in the silk gut powders from BmFibH-polyhedrin/H1/FGF-2 and BmFibH-H1/FGF-2 worms had greater p44/p42 phosphorylation activity than comparable untreated cells or cells treated with control silk gut powders from *w1-pnd* or BmFibH-polyhedrin worms (Fig. 3A).

Serum-starved NIH3T3 cells were cultured for 48 h in the presence of silk gut powders from the BmFibH-H1/FGF-2 and BmFibH-polyhedrin/H1/FGF-2 lines in serially increasing amounts, and cell proliferation was measured by WST-8 assay, which detects intracellular dehydrogenase (see the Methods section). As controls, cells cultured with the silk gut powders from *w1-pnd* and BmFibH-polyhedrin worms and fresh rhFGF-2 were similarly treated for the WST-8 assay (Fig. 3B). In the presence of silk gut powders from either BmFibH-H1/FGF-2 or BmFibH-polyhedrin/H1/FGF-2 lines, NIH3T3 cells proliferated in a dose-dependent manner to an extent that was comparable with the addition of rhFGF-2, whereas the cells did not proliferate in the absence of stimulation nor in the presence of control silk gut powders from *w1-pnd* or BmFibH-polyhedrin worms (Fig. 3B). When the silk gut powders (1 mg) from lines BmFibH-H1/FGF-2 or BmFibH-polyhedrin/H1/FGF-2 were added to the culture, the value obtained from the WST-8 assay became 1.77–2.00-fold greater than that of unstimulated cells and control silk gut powder-treated cells. The effect on NIH3T3 proliferation of the addition of approximately 10 ng of rhFGF-2 was estimated to correspond to 1 mg of silk gut powder from BmFibH-polyhedrin/H1/FGF-2 line. These results indicated that H1/FGF-2 in silk gut powder is active and released to stimulate the proliferation of NIH3T3 cells. However, it was still not clear whether, in the silk gut, polyhedra help to maintain the stability of H1/FGF-2 activity by protecting it from physical damage.

**Figure 4:**
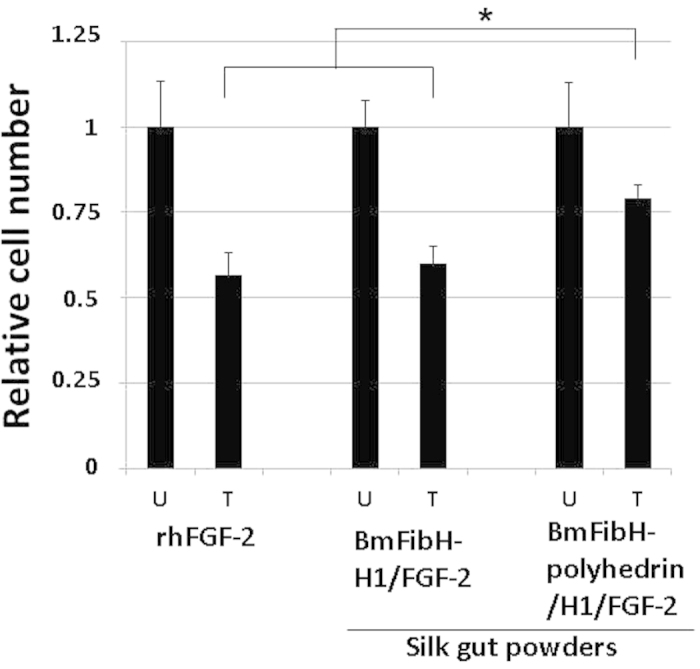
FGF-2 activity changes after incubation for one week at 25 °C. Silk gut powder samples (1 mg) from the BmFibH-polyhedrin/H1/FGF-2 and BmFibH-H1/FGF-2 lines suspended in PBS and diluted rhFGF-2 (10 ng) were placed on the inside filters of cell culture inserts. The samples in the cell culture inserts were air-dried and kept for one week at 25 °C (treated samples; column T). Untreated (column U) and treated samples in cell culture inserts were introduced into culture of serum-starved NIH3T3 cells. After 48 h of cultivation, cell counts were measured by WST-8 assay. Mean relative values of triplicate experiments are presented with standard deviations. *P < 0.01 *vs*. cells cultured with each sample of rhFGF-2 and silk gut powder from the BmFibH-H1/FGF-2 line after incubation for one week at 25 °C.

To assess the effect of polyhedra on the stability of H1/FGF-2, silk gut powders from BmFibH-H1/FGF-2 and BmFibH-polyhedrin/H1/FGF-2 lines, and fresh rhFGF-2 were maintained under aseptic conditions for one week at 25 °C and then applied to the assay for NIH3T3 cell proliferation. The activities of these two silk gut powders and rhFGF-2 were compared before and after the incubation for one week at 25 °C (Fig. 4). The cell proliferation-stimulating capacity of rhFGF-2 and BmFibH-H1/FGF-2 silk gut powder was strongly reduced (by 86.00% and 76.88% compared with the untreated sample, respectively) after incubation for one week at 25 °C (Fig. 4). However, the stimulatory effect of BmFibH-polyhedrin/H1/FGF-2 silk gut powder on NIH3T3 proliferation was less strongly reduced after incubation for one week at 25 °C (a reduction of 41.70% compared with the untreated sample; Fig. 4). These results demonstrated that polyhedra in the silk gut powder from transgenic silkworms effectively protect the biological activity of encapsulated FGF-2 against damage from storage at room temperature.

## Discussion

In the present study, we generated a transgenic *B. mori* line that has posterior silk glands producing FGF-2 immobilized by Cypovirus 1 polyhedra (Fig. 1 and 2). Previous studies revealed that polyhedrin can be massively expressed by utilization of a baculovirus vector in insect culture cells and it crystallizes readily^1^,^2^,^3^,^4^,^5^,^6^. To date, the details of the entire process of polyhedrin crystallization have not been elucidated. However, our previous X-ray crystallographic data revealed the three-dimensional structure of the polyhedra: polyhedrin autonomously forms trimers of linked polyhedrin by non-covalent interactions, tetrahedral clusters form by the assembly of four trimers, and many tetrahedral clusters assemble to form a cubic crystal^2^,^24^. Our recent attempts at *in vitro* crystallization of polyhedra by using monomer polyhedrin protein prepared either from bacterial expression or *in vitro* translation have failed despite attempts at varying conditions such as using excess polyhedrin and different autonomous crystallization conditions. Thus, polyhedrin crystallization occurring exclusively in living cells suggests the presence of specific cellular factors supporting polyhedrin crystallization in eukaryotic cells. Although polyhedrin was expressed in the posterior silk glands of the transgenic line from the 3rd to 6th day in the 5th instar, the polyhedral crystals increased to a dramatically larger size only on the 6th day (Fig. 1B), suggesting that the efficient formation and growth of polyhedra requires a massive accumulation of polyhedrin protein in eukaryotic cells.

The present study is an unprecedented demonstration of the encapsulation of an H1-tagged protein within crystallized polyhedra in living tissue as opposed to in cultured insect cells. However, the presence of biologically inactivated H1/FGF-2 after long-term incubation at 25 °C even in silk guts with polyhedra (Fig. 4) suggested that some H1/FGF-2 was not encapsulated within the polyhedra, and it was then impaired by hostile environmental damage. This was potentially attributable to an imbalance between the amount of polyhedra and H1/FGF-2 produced in the posterior silk glands, principally the insufficient formation of polyhedra to immobilize all of the coexpressed H1/FGF-2. Accordingly, to efficiently obtain polyhedron-encapsulated FGF-2 in posterior silk glands by reducing non-encapsulated FGF-2, the promoter activity for polyhedrin expression should be further optimized. A recent report demonstrated that the upstream 2.9 kbp sequence of *B. mori* HSP90 can act as a promoter/enhancer of gene expression to mediate expression levels of foreign proteins up to approximately ten-fold the maximum of that obtained using an actin promoter in silkworm individuals and insect culture cells^25^. Thus, it seems feasible to modify regulation by using optimized promoters to solve the problems of protein expression levels in living silkworm tissues.

The generation of a transgenic silkworm that produces a fusion protein of fibroin plus FGF-2 in posterior silk glands previously showed the difficulty of recovering active FGF-2 from spun silk fibre crystals because the fibroin-FGF-2 fusion protein in the silk fibre was significantly less biologically active^26^. However, our results unprecedentedly demonstrated the biological activity of polyhedron-encapsulated H1/FGF-2 produced in the posterior silk glands, which effectively maintained stable FGF-2 activity in a hostile environment, released active FGF-2, and stimulated NIH3T3 proliferation (Fig. 3,4). This is particularly significant when considering that the mean half-life of FGF-2 activity is only 7.6 h when administered in the body^27^. A better approach for FGF-2 use would be for the protein to be in an effective carrier that maintains and releases its activity. Therefore, it was suggested that silk guts or the related material including bioactive FGF-2-encapsulating polyhedra could be an effective carrier to be implanted and to control cell growth in the body. Recently, it was reported that baculoviruses can internalize within mammalian cells via stimulation of cell endocytosis or pinocytosis by the action of envelope glycoproteins and thereby functionally influence mammalian innate immune responses^28^. Additionally, cell culture systems include a grave concern of occasionally pathogenic serum. However, our system for the synthesis of polyhedron-encapsulated cell growth factors was developed using insect cell culture accompanied by mammalian foetal serum and a baculovirus vector system^1^,^2^,^3^,^4^,^5^,^6^. Taken together, our results suggest that polyhedron-encapsulated cell growth factor prepared from virus-free and serum-free systems might safely control cell proliferation *in vivo*.

Numerous studies have evaluated manufactured silk proteins as implantable materials mimicking extracellular matrices, especially for bone regeneration^16^,^17^,^18^,^19^. Proteinaceous silk materials can be physically and chemically manipulated, are biocompatible and are predictably biodegradable, as we have already used silk fibre as a suture thread for surgery. In addition, our results indicate that silk guts or the related materials have a slight effect on alleviating the damage to biological activity of potentially non-encapsulated FGF-2 from long-term incubation in 25 °C (Fig. 4). This finding suggests that the properties of silk proteins can prevent protein conformational changes or rapid protein degradation. The posterior silk glands that produce cell growth factor-encapsulating polyhedra were shown to maintain the biological activity of encapsulated FGF-2 more stably than the silk component (Fig. 4). The transgenic silkworms expressing these functional polyhedra in their posterior silk glands have potential advantages because production is easily scaled up without excessive cost, especially considering Japan’s industrial foundation in silk production.

It has been postulated that secretion of the expressed protein polyhedrin into the lumen of the tissue is achieved by utilizing the middle silk glands, which easily secrete foreign proteins^29^. Although it seems to be advantageous to prepare cocoon fibre consisting of polyhedra spun by transgenic silkworms, such an approach is not possible as polyhedrin can only crystallize in living eukaryotic cells. Thus, we propose that silk guts or the related materials with cells expressing FGF-2-encapsulating polyhedra should be utilized after the physical processing of malleable silk glands. By the traditional methods of the silk industry, for example by soaking in an appropriate concentration of dilute acetate or by air-drying, silk glands can be easily processed into silk guts (Fig. 1C) in preferential shapes suitable for use in the body. Our results indicate that the activities of polyhedron-encapsulated cell growth factors in silk guts are protected from physical damage during processing and long-term storage. Thus, future studies that develop processing approaches for silk glands will be necessary to expand the medical uses of silk guts incorporating cell growth factors in the body.

## Methods

### Formation of polyhedra and H1/FGF-2 in the posterior silk glands of transgenic silkworms

The *Bombyx mori* strain, *w1-pnd* was used to generate the transgenic silkworms (Supplementary Information). The Supplementary Methods describe the plasmid construction (Supplementary Fig. S1A) and generation of transgenic silkworm line BmFibH-polyhedrin/H1/FGF-2 (Supplementary Fig. S1B) with posterior silk glands expressing Cypovirus 1 polyhedra and human basic fibroblast growth factor tagged with the N-terminal H1-helix polyhedron-immobilization signal (H1/FGF-2)^3^. The BmFibH-H1/FGF-2 line expressing H1/FGF-2 in posterior silk glands under control of the fibroin H promoter is described in Supplementary Figure S1C. The oligonucleotides used for silkworm transgenesis are listed in Supplementary Table S2.

On the day presented in Fig. 1B, posterior silk glands were excised from 4th and 5th instar larvae of the BmFibH-polyhedrin line and mashed under glass cover slips. Crystallized polyhedra formed in the tissue were observed using an Olympus IX71 light microscope with an objective lens (20x). The images were acquired by using DP controller software.

Posterior silk glands from the BmFibH-polyhedrin line expressing only polyhedrin (Supplementary Fig. S1B), BmFibH-polyhedrin/H1/FGF-2, and BmFibH-H1/FGF-2 were excised, suspended in distilled water (40 mg wet weight/ml), sonicated in SDS-PAGE sample buffer (50 mM Tris-HCl, pH6.8, 100 mM dithiothreitol, 2% SDS, 0.1% bromophenol blue, 10% glycerol), and then boiled for 5 min at 100 °C. Commercial recombinant human FGF-2 (rhFGF-2; R&D Biochemicals, Inc.) and H1/FGF-2-encapsulating polyhedra prepared from Sf21 cell culture^3^ were treated as above. Denatured posterior silk gland proteins (7 or 20 μl), rhFGF-2 (10 ng) and H1/FGF-2- polyhedra from Sf21 (1 × 10^4^) were electrophoresed in a 12.5% gel with a Prestained XL ladder (Nacalai Tesque, Inc.) molecular marker, transferred onto a nitrocellulose membrane, and incubated overnight in an antibody buffer (20 mM Tris-HCl, 0.5 M NaCl, 0.05% Tween 20, pH 7.5) containing 1:3000 primary antibody. For the primary antibody, we used the polyhedrin antiserum described earlier^30^ and an anti-FGF basic antibody (Calbiochem) for the detection of polyhedrin and H1/FGF-2, respectively. Detection of specific proteins was performed by using buffer containing 1:3000 secondary antibody (Bio-Rad Laboratories, Inc.; goat-anti rabbit IgG for polyhedrin and goat-anti mouse IgG for H1/FGF-2) and the Peroxidase Stain DAB Kit, Brown Stain (Nacalai Tesque, Inc.) for staining.

Polyhedra from posterior silk glands of the BmFibH-polyhedrin and BmFibH-polyhedrin/H1/FGF-2 lines excised on the 6th day of the 5th instar were suspended in PBS, fixed on a glass-based dish by saturation with 4% paraformaldehyde for 15 min at room temperature, and incubated in a blocking solution, Blocking One (Nacalai Tesque, Inc.). Then, the dishes were saturated overnight with blocking solution containing 1:1000 anti-FGF basic antibody at room temperature, washed three times in PBS, and then incubated in the blocking solution containing 1:2000 Alexa Fluor 488-conjugated anti-mouse IgG (Molecular Probes, Inc) for 1 h at room temperature. After washing three times, polyhedra were examined by fluorescence microscopy with an Olympus IX71 light microscope with an objective lens (20x).

### Phosphorylation of p44/p42 mitogen-activated protein kinase (MAPK)

Posterior silk glands from the *w1-pnd* strain (a non-transgenic control), BmFibH-polyhedrin line (a transgenic control), BmFibH-polyhedrin/H1/FGF-2 line and BmFibH-H1/FGF-2 line on the 6th day of the 5th instar were dried and processed into silk guts. Next, the silk guts were lyophilized, crushed into fine powdery material (silk gut powder), and then kept for more than 24 h at −80 °C.

NIH3T3 cells were plated into a 6-well plate (Iwaki, Co) at a density of 1.0 × 10^5^ cells per well, cultured for 2 days, and treated by starvation in α-MEM medium for 24 h at 37 °C to synchronize the cell cycle before assays to evaluate the effects of silk gut powder. Silk gut powder (1 mg) suspended in the medium was placed on the inside filter of a Cell Culture Insert (BD Falcon), which was then introduced into the medium in wells. The cells were similarly treated with rhFGF-2 (10 ng) and polyhedron-encapsulated H1/FGF-2 from Sf21 (1 × 10^5^ cube). After 4 h of cultivation, the cells were washed in PBS twice and then dissolved in 100 μl of SDS-PAGE sample buffer. The cell lysates were harvested, sonicated, and heat-denatured. Samples (5 μg proteins) were electrophoresed, blotted onto a nitrocellulose membrane, and then analysed by immunoblotting as described above, incubating with either p44/p42 MAP Kinase Antibody or phospho-p44/p42 MAP Kinase (Thr202/Tyr204) Antibody followed by HRP-conjugated anti-rabbit IgG (Cell Signalling Technology, Inc.). Peroxidase activity was detected and visualized using ECL Western Blotting Detection Reagents (GE Healthcare).

### Proliferation assay of NIH3T3 cells

The proliferation of NIH3T3 cells was measured by using Cell Counting Kit-8, which detects intracellular dehydrogenase using a highly water-soluble tetrazolium salt, WST-8 (Dojindo Molecular Technologies, Inc.). For the WST-8 assay, cells were plated on 6-well plates at a density of 5 × 10^4^/well and cultured for 24 h. After starvation, the cells were cultured in the assay medium containing rhFGF-2 or with Cell Culture Inserts supplemented with silk gut powders from *w1-pnd*, BmFibH-polyhedrin, BmFibH-polyhedrin/H1/FGF-2 or BmFibH-H1/FGF-2 silkworms in the amounts presented in Fig. 3B. After incubation for 48 h at 37 °C under 5% CO_2_, Cell Counting Kit-8 solution was added to the cultures and incubated for 4 h at 37 °C under 5% CO_2_ according to the manufacturer’s instructions. Absorbance at 450 nm was determined by using a micro-plate reader Model 680 (Bio-Rad Laboratories, Inc.).

### Stability of rhFGF-2 and H1/FGF-2 in silk gut powder

To assess the influence of incubation for one week at 25 °C on FGF-2 activity, each silk gut powder (1 mg) suspended in PBS and diluted rhFGF-2 (10 ng) was placed on the inside filter of a Cell Culture Insert, and then air-dried under aseptic conditions. After being kept for one week at 25 °C, the Cell Culture Insert was introduced into an NIH3T3 culture. After 48 h of cultivation, cell counts were measured by Cell Counting Kit-8 as described above. Each assay was performed three times. Mean ± SD normalized relative activity was calculated for each experimental sample and significance was tested using one-way analysis of variance followed by Tukey’s test.

## Additional Information

**How to cite this article**: Kotani, E. *et al.* Cell proliferation by silk gut incorporating FGF-2 protein microcrystals. *Sci. Rep.* **5**, 11051; doi: 10.1038/srep11051 (2015).

## Supplementary Material

Supplementary Information
