# Supplementary material for: Cell proliferation by silk gut incorporating FGF-2 protein microcrystals
Source: Sci Rep. 2015 Jun 8;5:11051. doi: 10.1038/srep11051 (PMC4459171; doi:10.1038/srep11051)
Supplement: Supplementary Information [file srep11051-s1.pdf]

Supplementary Information for Cell proliferation by silk gut incorporating FGF-2 protein microcrystals by E. Kotani et al.

Eiji Kotani[1,2*], Naoto Yamamoto[1], Isao Kobayashi[3], Keiro Uchino[3], Sayaka Muto[1], Hiroshi Ijiri[1], Junji Shimabukuro[1], Toshiki Tamura[3], Hideki Sezutsu[3] & Hajime Mori[1,2*]

[1]Department of Applied Biology, Kyoto Institute of Technology, Sakyo-ku, Kyoto 606-8585, Japan [2]Insect Biomedical Centre, Kyoto Institute of Technology, Sakyo-ku, Kyoto 606-8585, Japan [3]Transgenic Silkworm Research Unit, National Institute of Agrobiological Sciences, Tsukuba, Ibaraki 305-8634, Japan

*Corresponding authors: kotani@kit.ac.jp, hmori@kit.ac.jp

Supplementary Methods

Silkworm strain and cultured cells. A non-diapausing *Bombyx mori* strain, *w1-pnd,* which has a non-pigmented skin trait during the first instar and the adult eyes, was used for the generation of transgenic silkworms. Silkworm larvae were reared on an artificial diet (Aseptic Sericulture System Laboratory in Kyoto) at 25°C under aseptic conditions.

The NIH3T3 mouse embryonic fibroblast cell line was cultured in D-MEM (high glucose) with L-glutamine, phenol red and sodium pyruvate (Wako Pure Chemical Industries, Ltd.) supplemented with 10% foetal bovine serum (Biological Industries, Ltd.) at 37°C under 5% CO_2.

Construction of vectors and generation of transgenic silkworms Plasmids used to generate the transgenic silkworms (Supplementary Fig. S1A) were confirmed by DNA sequencing. The oligonucleotide sequences used in this study are summarized in Supplementary Table S2. To construct the pBacMCS[A3KMO,UAS-polyhedrin-SV40] vector, a DNA fragment with the complete open reading frame of Cypovirus 1 polyhedrin[1,2] was amplified from the viral sequence, digested by the restriction enzyme *Bln* I, and inserted into the *Bln* I site downstream of the UAS in the plasmid

pBacMCS[A3KMO,UAS][8]. To construct the pBacMCS[UAS-H1/FGF-2,3xP3-egfp] vector, the sequence of the human basic fibroblast growth factor fused with the N-terminal H1-helix (polyhedron-encapsulation signal), H1/FGF-2, was amplified and inserted into the *Bln* I site downstream of the UAS sequence in the plasmid pBacMCS[UAS, 3xP3-egfp][3,14]. Plasmids were purified by using a Plasmid Midi-Prep Kit (QIAGEN) in accordance with the manufacturer's instructions.

To generate transgenic silkworms, we performed microinjection as previously described[9,10]. Donor and helper plasmids for the production of transposase were injected into embryos at the pre-blastoderm stage[9,10]. A transgenic silkworm line possessing the UAS-polyhedrin gene was generated by using pBacMCS[A3KMO,UAS-polyhedrin-SV40] and screening the G1 first instar larvae for the trait from the vector-derived marker, *kynurenine 3-mono oxygenase* (*KMO*) -specific skin colour. Other transgenic silkworms possessing the UAS-H1/FGF-2 gene were identified by the trait of vector-derived EGFP expression driven by 3xP3 at the late G1 embryonic stage. The transgenesis of these genes was confirmed by an inverse PCR method as follows. Genomic DNA from the silk glands of G2 fifth instar larvae was purified by standard phenol treatment after overnight incubation with proteinase K, digested with *Sau*3AI, and circularized by overnight ligation at 16°C using Ligation

high version 2 (Toyobo, Co.). DNA fragments were amplified from the circularized template by PCR amplification from both upstream and downstream ITRs of the *piggyBac* sequence (Supplementary Table S1). Transgenic lines carrying the genes for polyhedrin (UAS-polyhedrin line) and H1/FGF-2 (UAS-H1/FGF-2 line) were further established by repeated sib mating of selected individuals with the trait of *KMO*-specific skin or EGFP expression in the eyes, respectively. Next, individuals of the UAS-polyhedrin transgenic line were mated with the previously established GAL4-driver line, BmFibH-GAL4[15], which has the marker 3xP3-controlled DsRed and expresses GAL4 protein under control of the fibroin-heavy chain (fibroin H) long promoter. Consequently, we obtained a BmFibH-polyhedrin line expressing polyhedrin under control of the fibroin H promoter (Supplementary Fig. S1B). Then, the BmFibH-polyhedrin line was mated with the UAS-H1/FGF-2 line to generate the BmFibH-polyhedrin/H1/FGF-2 line (Supplementary Fig. S1B), which produces both polyhedrin and H1/FGF-2 in its posterior silk glands. The BmFibH-GAL4 line was mated with the UAS-H1/FGF-2 line to generate the BmFibH-H1/FGF-2 line, which produces only H1/FGF-2 protein in its posterior silk glands (Supplementary Fig. S1C). Homogeneous BmFibH-polyhedrin/H1/FGF-2 and BmFibH-H1/FGF-2 lines were

established through more than five generations of repeated sib mating and isolation using each marker trait.

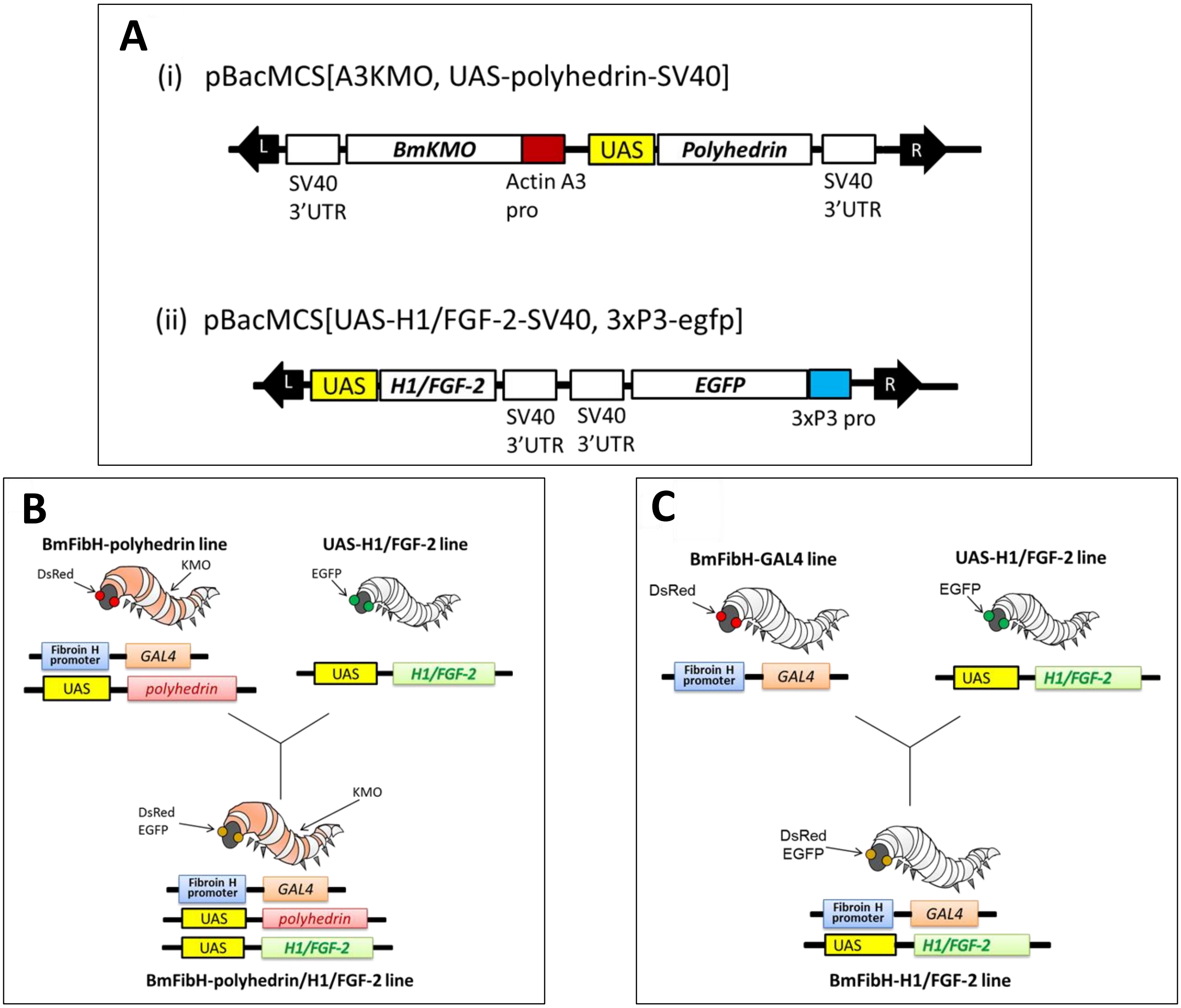

Supplementary Figure S1 | Generation of the transgenic silkworms. (A) Schematic representation of the vectors used to generate the transgenic silkworms. The plasmids, pBacMCS[A3KMO,UAS-polyhedrin-SV40] encoding *B. mori* kynurenine 3-mono oxygenase (*BmKMO*) plus polyhedrin (i) , and pBacMCS[UAS-H1/FGF-2-SV40/3xPs-GFP] encoding EGFP plus H1/FGF-2 (ii) were used to generate the transgenic lines termed UAS-polyhedrin and UAS-H1/FGF-2, respectively. The *piggyBac* right and left ITRs (L and R) are indicated in black; UAS, in yellow; *B. mori* actin A3 promoter, in red; 3xP3 promoter, in blue. (B) Schematic flow for generation of the BmFibH-polyhedrin/H1/FGF-2 line: the BmFibH-polyhedrin line was mated with the UAS-H1/FGF-2 line to generate the BmFibH-polyhedrin/H1/FGF-2 line with eyes concurrently expressing EGFP and DsRed plus *KMO*-specific skin. The drawing was made by E.K. and N.Y. using Adobe Illustrator and Power Point. (C) Schematic flow for generation of the BmFibH-H1/FGF-2 line: the BmFibH-GAL4 line was mated with the UAS-H1/FGF-2 line to generate the BmFibH-H1/FGF-2 line with eyes concurrently expressing EGFP and DsRed. The drawing was made by E.K. and N.Y. using Adobe Illustrator and Power Point.

Fig. 2A, upper

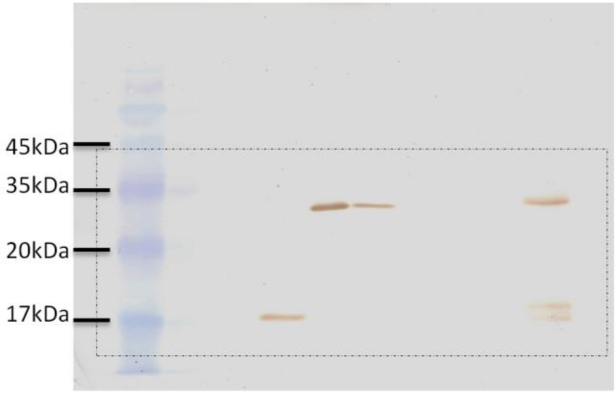

Fig. 2A lower

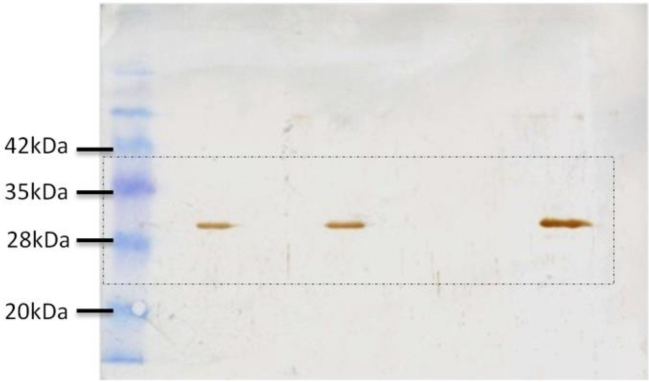

Supplementary Figure S2 | Original gel images of immunoblotting .

Fig. 3A, upper

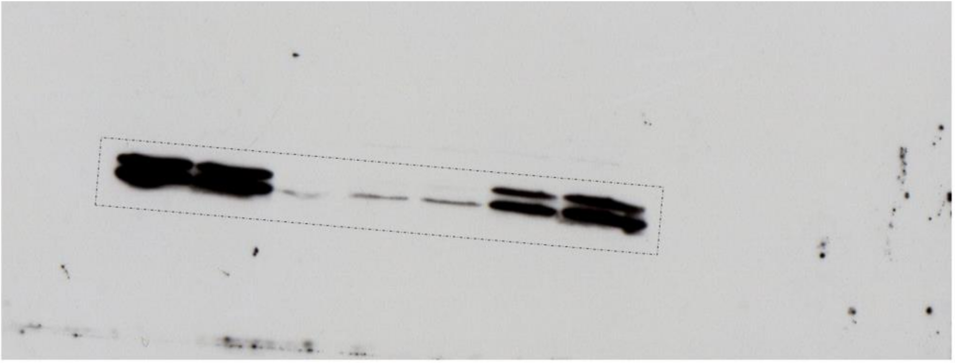

Fig. 3A, lower

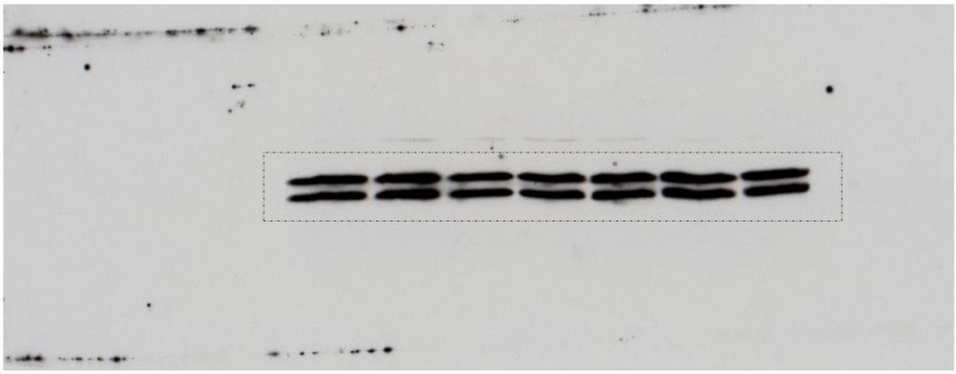

Supplementary Figure S2 | Original gel images of immunoblotting (continued).

Supplementary Table S1 | Genomic sequences at the borders of the transgene insertion regions of the transgenic *B. mori* lines. Genomic DNA was isolated from the transgenic lines, UAS-polyhedrin and UAS-H1/FGF-2, and the sequences at the borders of the transgene insertions were investigated by an inverse-PCR method and database analysis using the KAIKOBLAST database (http://kaikoblast.dna.affrc.go.jp). The border sequences are listed with the information of the chromosome and clone name. The consensus sequence TTAA at the border of the *piggyBac*-driven transgene is indicated in bold.

Lines	Sequence at the border of the transgene	Chromosome no. and (clone name)
UAS-polyhedrin	GTACATTTTTTGACTTC**TTAA**(Transgene)**TTAA**AAGACGGACACAAAAAC	Chromosome 6 (Bm_scaf78)
UAS-H1/FGF-2	CTACTTGGAATCCTTAG**TTAA**(Transgene)**TTAA**GCGTATAACGCCATCCC	Chromosome 11 (Bm_scaf24)

Supplementary Table S2 | Oligonucleotide primers used in this study. Authentic coding sequences of polyhedrin, FGF-2 and *piggyBac* are underlined, and restriction enzyme sites are shown in bold.

Primers	Sequence (5'-3')
(for cloning)	
polyhedrin 5': *Bln* I	AAA**CCTAGG**ATGGCAGACGTAGCAGGAACAAGTAACC
polyhedrin 3': *Bln* I	AAA**CCTAGG**CTACTGACGGTTACTCAGAGCTACTCCA
FGF-2 5': *Bln* I	TTT**CCTAGG**ATGGCAGACGTAGCAG
FGF-2 3': *Bln* I	TTT**CCTAGG**TCAGGCTTAGCAGACATTG
(for inverse-PCR)	
piggyBac right ITR 1: 5'	CGCATGATTATCTTTAACGTACGTCAC
piggyBac right ITR 2: 3'	GGGGTCCGTCAAAACAAAACATC
piggyBac left ITR 1: 5'	GAGTCTCTGCACTGAACATTGTCA
piggyBac left ITR 2: 3'	ATCAGTGACACTTACCGCATTGACA
